# Enhancing leaf disease classification using GAT-GCN hybrid model

**DOI:** 10.3389/fpls.2025.1569821

**Published:** 2025-08-06

**Authors:** Shyam Sundhar, Riya Sharma, Priyansh Maheshwari, Suvidha Rupesh Kumar, T. Sunil Kumar

**Affiliations:** ^1^ School of Computer Science and Engineering, Vellore Institute of Technology (VIT), Chennai, Tamil Nadu, India; ^2^ Department of Electrical Engineering, Mathematics and Science, University of Gävle, Gävle, Sweden

**Keywords:** leaf disease detection, Graph Convolution Networks, Graph Attention Networks, hybrid model, apple leaf, sugarcane leaf, potato leaf

## Abstract

Agriculture plays a critical role in the global economy, providing livelihoods and ensuring food security for billions. Progress in agricultural techniques has helped boost crop yield, along with a growing need for precise disease monitoring solutions. This requires accurate, efficient, and timely disease detection methods. The research presented in this paper addresses this need by analyzing a hybrid model built using Graph Attention Network (GAT) and Graph Convolution Network (GCN) models. The integration of these models has witnessed a notable improvement in the accuracy of leaf disease classification. GCN has been widely used for learning from graph-structured data, and GAT enhances this by incorporating attention mechanisms to focus on the most important neighbors. The methodology incorporates superpixel segmentation for efficient feature extraction, partitioning images into meaningful, homogeneous regions that better capture localized features. The robustness of the model is further enhanced by the edge augmentation technique. The edge augmentation technique in the context of graph has introduced a significant degree of generalization in the detection capabilities of the model as analyzed on apple, potato, and sugarcane leaves. To further optimize training, weight initialization techniques are applied. The hybrid model is evaluated against the individual performance of the GCN and GAT models and the hybrid model achieved a precision of 0.9822, recall of 0.9818, and F1-score of 0.9818 in apple leaf disease classification, a precision of 0.9746, recall of 0.9744, and F1-score of 0.9743 in potato leaf disease classification, and a precision of 0.8801, recall of 0.8801, and F1-score of 0.8799 in sugarcane leaf disease classification. The results indicate that the model is effective and consistent in identifying leaf diseases in plants.

## 1 Introduction

**Figure 1:**
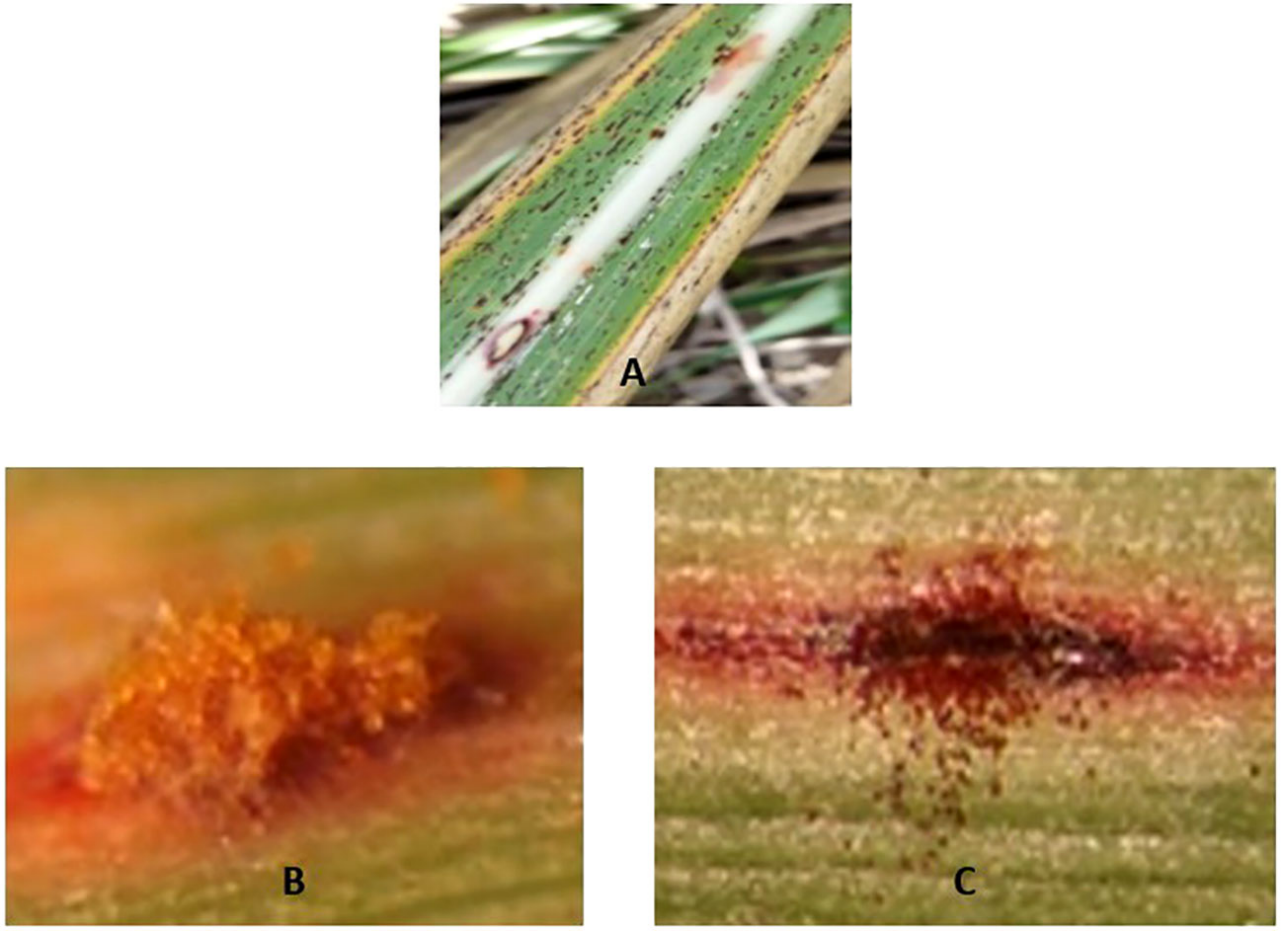
Visual illustration of rust disease in leaves **(A)**. Rust disease in sugarcane leaf **(B)**. Orange rust pustules **(C)**. Brown rust pustules (Simões et al., 2023).

The detection of plant diseases is important in agriculture, significantly impacting crop yield and overall productivity. Plant disease leads to biological and economic losses that leave millions of people starving and undernourished (Oerke, 2006). With the adoption of different agricultural practices and the intensification of climatic changes, the prevalence of crop diseases has increased. This, in turn, has made it a necessity to develop efficient and accurate leaf disease detection methods. Traditional approaches often rely on manual inspections by farmers, which can be time-consuming and subjective, leading to delays in disease management and substantial crop losses. In response to the evolving demands of modern agriculture, there is a need for effective disease detection strategies. Plant diseases, especially those affecting leaves, can often be identified by visible changes such as discoloration or shrinkage (Agrios, 2005). Rust is one of the most common and visually identifiable leaf diseases. A sample image from the dataset showing a leaf affected by rust is presented in **Figure 1A**. There are variations of the rust disease with respect to color as show in **Figures 1B, C**.

These visual cues have paved the way for identification using computer vision methods. The integration of machine learning (ML) and advanced computer vision technologies offers a promising solution (Jafar et al., 2024), enabling rapid analysis of leaf images and facilitating timely interventions essential for maintaining healthy crops. Recent advances in ML and deep learning (DL) have significantly improved the efficiency of plant disease detection. For example, computer vision algorithms have shown high performance in detecting tomato leaf diseases (Harakannanavar et al., 2022), and deep learning models such as AlexNet have demonstrated potential in identifying olive leaf diseases (Alruwaili et al., 2019).

Many of the existing methods are limited to binary classification tasks (i.e., Healthy vs. Non-Healthy), restricting their applicability in real-world scenarios where distinguishing between multiple disease types is critical. Furthermore, environmental variations such as lighting, background noise, and plant physiology pose further challenges in generalizing DL models to diverse agricultural settings. The proposed work is a small step toward addressing such limitations. This paper proposes a hybrid model built using the Graph Convolution Network (GCN) and the Graph Attention Network (GAT) for multiclass classification of leaf diseases. By combining the spatial feature learning capabilities of GCN with the dynamic feature prioritization mechanism of GAT, the model captures both structural and contextual nuances of disease symptoms more effectively. Superpixel-based image segmentation is employed to preserve local structures and enhance disease localization. In addition, the edge augmentation and optimized weight initialization techniques are incorporated to improve the robustness of the model. To demonstrate the generalized behavior of the proposed model, experiments were conducted on three diverse datasets, namely Sugarcane Leaf Disease, Apple Leaf Disease, and Potato Leaf Disease. These dataset contain leaf images presenting distinct visual and pathological characteristics, leading to the comprehensive evaluation of the performance and adaptability of the model.

The GCN and GAT architectures have been effectively applied in various other domains. In the field of drug discovery (Sun et al., 2020), the GCN model the molecular interactions, in 3D shape analysis (Wei et al., 2020), view-based GCN aggregate multi-view features, and in point cloud segmentation (Wang et al., 2019), Graph Attention Convolution (GAC) dynamically adjusts receptive fields to capture fine-grained structures. Inspired by these applications, the proposed work adapts GCN-GAT architectures for the nuanced detection of leaf disease. It takes advantage of the ability of GCN to model spatial correlations among segmented leaf regions and uses attention mechanism of GAT to prioritize patterns indicative of disease symptoms. This integration aims to enhance classification accuracy and robustness, particularly under varying environmental conditions.

In summary, the major contributions of this research include:

- GCN-GAT hybrid model that integrates the spatial feature-capturing ability of GCN with the feature prioritization strengths of GAT.
- Superpixel-based segmentation, edge augmentation, and tailored weight initialization to improve model robustness and localization.
- Evaluation on three diverse datasets (apple, potato, and sugarcane leaves) to demonstrate model generalize behavior and adaptability.
- Comparative experiments with standalone GCN and GAT models to validate the efficacy of the proposed hybrid architecture.
- Performance assessment using key metrics such as F1-score, accuracy, precision, recall, cross-entropy loss, and confusion matrix.

The remainder of this paper is structured as follows. Section 2 presents a literature review and discusses prior research in the field. Section 3 describes the dataset, the preprocessing steps applied, the proposed methodology, and the loss and evaluation metrics used in the experiments. Section 4 presents the results and analysis, comparing the performance of the proposed model with baseline methods. Finally, Section 5 concludes the paper, summarizes the key findings, and outlines potential directions for future research.

## 2 Review of prior findings

This literature review aims to provide an overview of the recent advancements in the realm of plant leaf disease detection. It examines a range of approaches that involves traditional machine learning approaches, state-of-the-art deep learning architectures that integrate multiple techniques. By synthesizing findings from key studies, the review also identifies research gaps and highlights areas that require further exploration, particularly focusing on improving model adaptability and generalization, as well as enhancing disease detection systems in diverse and dynamic agricultural environments

(Oo and Htun, 2018) presented a detection and classification system for four plant leaf diseases, including Cercospora Leaf Spot, Bacterial Blight, Powdery Mildew, and Rust. It involves image preprocessing, segmentation, and feature extraction using GLCM and LBP techniques. Various machine learning models were used, with the highest accuracy of 98.2% achieved by SVM. A comparative evaluation was conducted using classifiers such as SVM, KNN, and ensemble methods. The use of texture feature extraction through GLCM and classification enhanced using LBP by leveraging both statistical and structural characteristics. However, the study relies on feature extraction techniques that lack structural relationships among the features of the leaves. (Karlekar and Seal, 2020) addressed issues related to leaf segmentation and disease classification with a two-part approach: extracting leaf images through a specific method and employing a deep learning model called SoyNet for soybean disease classification. The model achieved an accuracy of 98.14%. An Integrated Pest Management (IPM) technique ensured the segmentation of leaf regions, even in complex backgrounds, focusing the model on relevant areas. Additional testing on the large Plant Disease Database (PDDB) dataset, which contains 16 disease categories, aimed to improve generalization across various types of disease. However, since the model was tested on one dataset only, its adaptability to new data remains limited.

(Wang et al., 2022) proposed a plant disease recognition model integrating visual and textual data through feature decomposition and GCNs. The model was evaluated on datasets with both uniform and non-uniform severity levels. Traditional networks like ResNet18 performed well on uniform data, while feature decomposition improved results for more complex data. An accuracy of 97.62% was reported with precision, sensitivity, and specificity values of 92.81%, 98.54%, and 93.57%, respectively, demonstrating the efficacy of multimodal approaches. It was observed that the two-layer GCN outperformed both singlelayer and three-layer models by optimally extracting features from the graph structure. However, the study used a static graph structure, which may not fully capture the dynamic nature of disease progression. Dynamic graph models could be explored to better represent changing disease information. A review by (Lu et al., 2021) focused on the application of deep learning methods, specifically CNN for the classification of plant leaf disease. Techniques like segmentation, data augmentation, and transfer learning were examined to overcome challenges such as limited datasets and robustness issues. Transfer learning-based CNN models achieved an accuracy of 95%. The review also explored image segmentation techniques like watershed segmentation, Otsu’s thresholding, and K-means clustering to isolate leaves from complex backgrounds, an often overlooked aspect in related research. Although CNN models are effective at capturing pixel-level features, they are limited in modeling the relational and structural characteristics inherent in disease patterns.

(Rao et al., 2024) presented a study combining CNN and GCN to improve plant disease classification. The integrated model achieved an accuracy of 99%, surpassing traditional models like DeepPlantNet (98%), ensemble models (91%), and transfer learning approaches (95%). By incorporating image-based features and plant connectivity, the model provided better contextual awareness and higher accuracy in disease classification. However, this approach requires significant computational resources and longer training times compared to a standalone CNN or GCN model. In addition, its performance heavily depends on the availability of large and balanced datasets. (Peng and Wang, 2022) introduced a new system for automatic identification, localization, and detection of leaf diseases using an image retrieval approach that incorporates object detection and deep metric learning. They first enhanced the YOLOv algorithm to improve the detection of small objects, allowing a more accurate extraction of leaf objects. The system also integrates classification recognition with metric learning, enabling the model to jointly learn both categorization and similarity measurements, hence enhancing the performance of existing image classification models. This approach allows for the addition of new disease types without the need for retraining. Experimental results on three publicly available leaf disease datasets demonstrated the effectiveness of the proposed system. This study demonstrates the application of the system to practical use cases in intelligent agriculture, such as crop health monitoring and nutrition diagnosis. However, there is an urgent need to improve the scalability and adaptability of the system to handle a broader range of plant species and environmental variations, ensuring its robustness in diverse agricultural settings.

(Rathore and Prasad, 2020) proposed an automatic method for the detection of diseases in rice plants using a CNN model. The model classified rice images into “Healthy” and “Leaf Blast” categories with an accuracy of 99.61% using a dataset of 1,000 images. Data augmentation techniques, including random rotation, shifting, flipping, and cropping, were used to expand the dataset and improve the generalization of the model. Although effective, the applicability of the model is limited as it classifies only two classes, namely healthy and leaf blast, thus restricting its use in detecting a broader range of rice diseases. (Roy et al., 2023) introduced an advanced plant disease segmentation method for precision agriculture using optimal dimensionality reduction with fuzzy C-means clustering and deep learning. The approach focused on the segmentation of rice leaf regions and the classification of diseases with high accuracy. The model used CNN models and achieved an accuracy of 99.61% on a dataset containing 1,000 images. Data augmentation techniques were used to increase performance. However, similar to (Rathore and Prasad, 2020), the model was designed to classify only two categories: healthy and leaf blast, consequently limiting its generalization to a wider variety of diseases and pests.

(Liu et al., 2020) proposed a novel approach for the identification of grape leaf diseases using an improved convolution neural network (CNN). The study focused on six major grape leaf diseases—anthracnose, brown spot, mites, black rot, downy mildew, and leaf blight—that cause significant economic losses in the grape industry. To address this challenge, the authors developed a dataset of 107,366 grape leaf images through image enhancement techniques, utilizing 4,023 fieldcollected images and 3,646 images from public datasets. The method incorporates an Inception structure to enhance multi-dimensional feature extraction and introduces a dense connectivity strategy to promote feature reuse and propagation. The proposed deep learning model, named DICNN, was trained from scratch and achieved an overall accuracy of 97.22% on a hold-out test set. Compared to GoogLeNet and ResNet-34, DICNN improved recognition accuracy by 2.97% and 2.55%, respectively. Although the model demonstrates strong performance, a key research gap remains in its ability to generalize across different crops and diverse environmental conditions (Bansal et al., 2021) developed a deep learning-based approach for the detection of apple leaf diseases. The dataset consists of 3,642 images divided into four classes, namely apple scab, apple cedar rust, multiple diseases, and healthy leaves. Pre-processing techniques such as flipping, rotation, and blurring were applied, and images were resized to 512x512 pixels. The authors used pre-trained models, including DenseNet121, EfficientNetB7, and EfficientNet NoisyStudent, with an ensemble model achieving a maximum accuracy of 96.25%. The ensemble approach effectively reduced variance and improved classification accuracy. However, relying solely on pre-trained CNN models may not fully capture the intricate relationships between different parts of a leaf, especially in cases involving multiple diseases.

**Table 1** summarizes recent key studies in plant leaf disease detection, highlighting their methodologies, datasets, performance metrics, and research gaps. Although considerable advances have been made using deep learning and hybrid models, key challenges remain. Many models are tailored to specific species or datasets and lack the generalizability needed for broader agricultural applications. Realworld variability, such as lighting, leaf orientation, and background clutter, continues to affect model performance. Moreover, scalability to multispecies or multilabel classification, real-time detection of subtle symptoms, and robust performance under constrained data scenarios are areas needing further work. Although attention mechanisms and graph-based methods like GCNs and GATs have shown promise, they often fail to capture both spatial relationships and contextual dependencies.

**Table 1:** Summary of recent research on plant disease detection and classification.

Author(s) and year	Methodology	Dataset	Performance
(Luo et al., 2021)	Optimized multi-scale fusion network with enhanced ResNet backbone, pyramid and dilated convolutions	Original and preprocessed apple leaf disease datasets	Classification accuracy: 94.24% (original), 94.99% (preprocessed)
(Dai et al., 2022)	Hybrid graph representation learning framework GraphCDA combining GCN and GAT, with Bayesian surrogate model for automated model selection	Disease-associated circRNAs data	Improved prediction via adaptive model selection (quantitative metrics not specified)
(Khan et al., 2022)	Two-stage apple disease detection: transfer learning with Xception for classification, Faster-RCNN for localization	Apple disease images	88% classification accuracy
(Shoaib et al., 2022)	Inception Net-based classification and Modified U-Net semantic segmentation for tomato plant disease detection	Dataset of 18,161 segmented and non-segmented tomato leaf images	Modified U-Net accuracy 98.66%, IoU 98.5, Dice 98.73; InceptionNet accuracy 99.95% (binary), 99.12% (six-class)
(Liu and Wang, 2023)	Tomato disease detection using prior knowledge attention mechanism and multi-scale features (PKAMMF), new feature fusion and prediction layers, Adaptive Structured IoU loss	Self-built tomato disease dataset	mAP of 91.96%, 3.86% improvement over baselines
(Mahum et al., 2023)	Improved deep learning classification of potato leaf diseases using pre-trained Efficient DenseNet with additional transition layer and reweighted cross-entropy loss	Potato leaf disease dataset	Accuracy of 97.2%
(Wang et al., 2024)	Attention mechanisms with CBAM and multi-scale feature fusion via BiRepGFPN replacing PAFPN in YOLOv6 for tomato leaf disease detection	PlantDoc and tomato leaf disease datasets	Significant improvements in mAP, precision, recall, and F1-score
(Bera et al., 2024)	PND-Net: GCN on top of CNN with spatial pyramidal pooling for plant nutrition deficiency and disease classification	Banana, coffee nutrition deficiency, potato disease, and PlantDoc datasets	Accuracies: 90.00% (banana), 90.54% (coffee), 96.18% (potato), 84.30% (PlantDoc)

To address these gaps, the proposed hybrid GCN-GAT model offers improved generalization and spatial relational learning, enabling robust and accurate detection of leaf diseases. The overarching research gaps identified in the literature can be summarized as follows.

- Limited generalization of existing architectures across diverse plant species and disease types hinders scalability.
- Inadequate modeling of spatial and relational dependencies between leaf regions, reducing detection precision.
- Performance degradation under real-world environmental conditions such as lighting changes, occlusions, and background noise.

## 3 Methodology

### 3.1 Dataset description

The hybrid model is analyzed using three different leaf disease datasets, namely: Sugarcane leaf disease dataset (Sankalana, 2022), potato leaf disease data set (Putra, 2020), and apple leaf disease data set (Antor, 2020), each capturing a diverse set of images taken under various lighting conditions.

#### 3.1.1 Sugarcane Leaf Disease Dataset

The Sugarcane Leaf Disease Dataset consists of 2521 RGB images of sugarcane leaves, collected manually from various regions of Maharashtra, India. The images are categorized into five distinct classes, including 522 images of healthy leaves, 462 images showing mosaic symptoms, 518 images of red rot disease, 514 images of rust and 505 images of yellow disease.

#### 3.1.2 Potato Leaf Disease Dataset

The Potato Leaf Disease Dataset contains 1200 RGB images of potato leaves, classified into three types of disease: Early Blight, Late Blight, and Healthy leaves with each class having 300 samples of leaves.

#### 3.1.3 Apple Leaf Diseases Dataset

The Apple Leaf Diseases Dataset is a comprehensive collection of 480 RGB images designed to support the identification of various foliar diseases in apple trees. The images in this dataset are categorized into three primary disease types: Apple Black Rot (170 samples), Cedar Rust (160 samples), and Apple Scab (150 samples).

For all datasets, the images were split into training and testing sets with an 80–20 ratio to ensure a balanced representation of classes and effective model evaluation and has been tabulated in **Table 2**.

**Table 2:** Distribution of training and testing images across leaf disease classes.

Class	Training	Testing	Total per class
Sugarcane _Healthy	418	104	522
Sugarcane _Mosaic	369	93	462
Sugarcane _Red Rot	414	104	518
Sugarcane _Rust	411	103	514
Sugarcane _ Yellow	404	101	505
Potato _Early Blight	300	100	400
Potato _Late Blight	300	100	400
Potato _Healthy	300	100	400
Apple _Scab	120	30	150
Apple _Black Rot	136	34	170
Apple _Cedar Rust	128	32	160

### 3.2 Preprocessing

To ensure the efficiency of the model, several preprocessing techniques were applied to the dataset. Initially, to standardize the input size, all images were resized to 128x128 pixels. This resizing step ensures uniformity across the dataset, which is essential for consistent model performance. Furthermore, to facilitate faster convergence during training, the pixel values of the images were normalized to the range [−1,1], using a mean of 0.5 and a standard deviation of 0.5, as shown in Equation 1. This normalization procedure helps stabilize the training process and accelerates convergence. A batch size of 32 was selected for training, balancing computational efficiency and the stability of gradient updates.


(1) $$x_{\text{normalized}}=\frac{x/255-\mu }{\sigma }=2\cdot (\frac{x}{255}-0.5)$$

- *x* is the original pixel value in the range [0,255].
- *µ* = 0.5 and *σ* = 0.5 are the mean and standard deviation used for normalization.
- This transformation scales the input to the range [−1,1] to improve training performance.

In addition to resizing and normalizing, superpixel segmentation was employed to enhance feature extraction. The Simple Linear Iterative Clustering (SLIC) algorithm (Achanta et al., 2012) was used to partition each image into perceptually meaningful regions, or superpixels, based on color similarity and spatial proximity. The mathematical formulation of the distance metric used in SLIC is presented in Equation 2.


(2) $$D=\sqrt{d_{c}^{2}+{\left(\frac{m}{S}\right)}^{2}d_{s}^{2}}$$

- *D* is the combined distance used to assign pixels to superpixels.
- *d_c_* is the color distance in the CIELAB space.
- *d_s_* is the spatial distance between the pixel coordinates.
- *m* is the compactness parameter controlling the shape regularity of superpixels.
- *S* is the grid interval, representing the approximate spacing between the superpixel centers.

**Figure 2:**
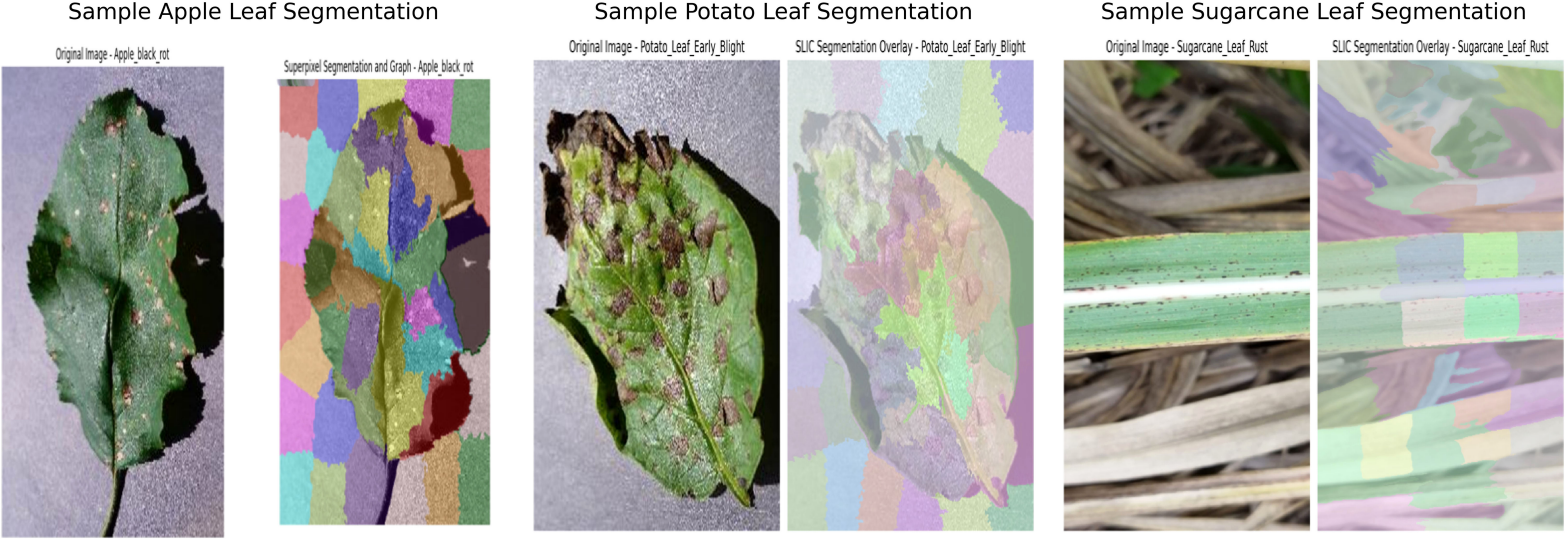
Preprocessed images of apple, potato, and sugarcane leaves using superpixel segmentation.

This segmentation technique allows graph-based models to focus on localized regions of the image, which is particularly beneficial for detecting fine-grained textures and structures indicative of leaf diseases. Based on experimental evaluation, the number of superpixels was set to 50, which provided a detailed and balanced representation of the image regions. Lower values such as 20 captured insufficient features, while higher values such as 100 led to overfitting. Some representative examples of segmented leaf images are shown in **Figure 2**.

Following segmentation, a Region Adjacency Graph (RAG) was constructed to model the spatial relationships between adjacent superpixels. In this graph, each superpixel is represented as a node, and the edges encode adjacency between neighboring regions. The mean RGB color of each superpixel was extracted as a node feature, providing a compact yet informative representation of the visual content. The encoded adjacency information enables the graph-based model to learn spatial relationships more effectively, and in turn improvising their ability to capture complex visual patterns. These RAG features, along with the corresponding class labels, were stored in pickle files and used during the training phase of the model.

### 3.3 Methodology

#### 3.3.1 Machine Learning

Machine Learning (ML) is a subset of artificial intelligence (Samuel, 1959) that enables machines to learn from data and make predictions or decisions without being explicitly programmed. It involves developing algorithms that iteratively improve their performance by minimizing a loss function based on experience or data.

#### 3.3.2 Deep Learning

Deep Learning (DL) is a specialized branch of machine learning that uses multi-layered neural networks (Hinton et al., 2006) to automatically learn representations from raw data. This hierarchical feature learning enables models to capture complex patterns and is particularly effective in tasks such as image classification and natural language processing.

#### 3.3.3 Graph Convolution Network

**Figure 3:**
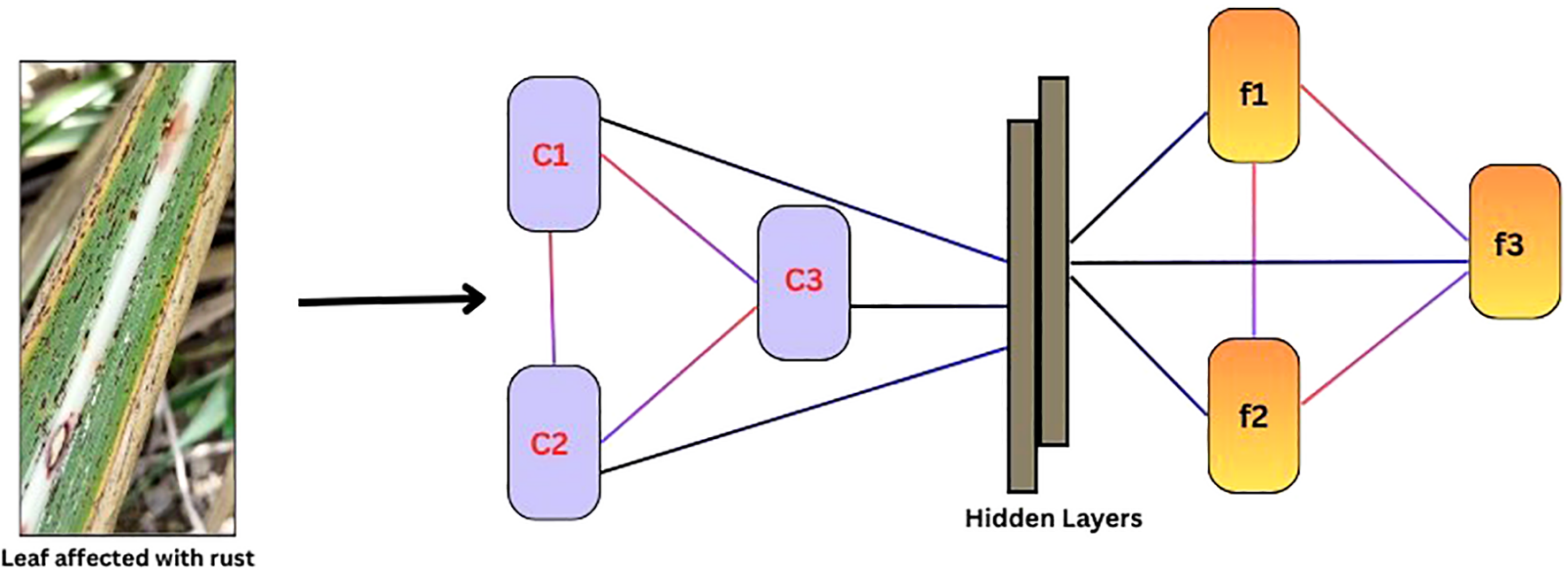
Schematic representation of the Graph Convolution Network (GCN) architecture.

A Graph Convolution Network (GCN) is a neural network designed for graph-structured data (Kipf and Welling, 2016). It captures node-level features and local relationships between neighboring nodes by aggregating information from them. The core mechanism of GCNs involves message passing, where each node updates its representation based on the features of its neighbors. This is achieved through feature propagation, where nodes receive information from connected neighbors, followed by graph convolution, where aggregated features are transformed through a weighted sum. A simple structure of a GCN is depicted in (**Figure 3**).

#### 3.3.4 Graph Attention Network

**Figure 4:**
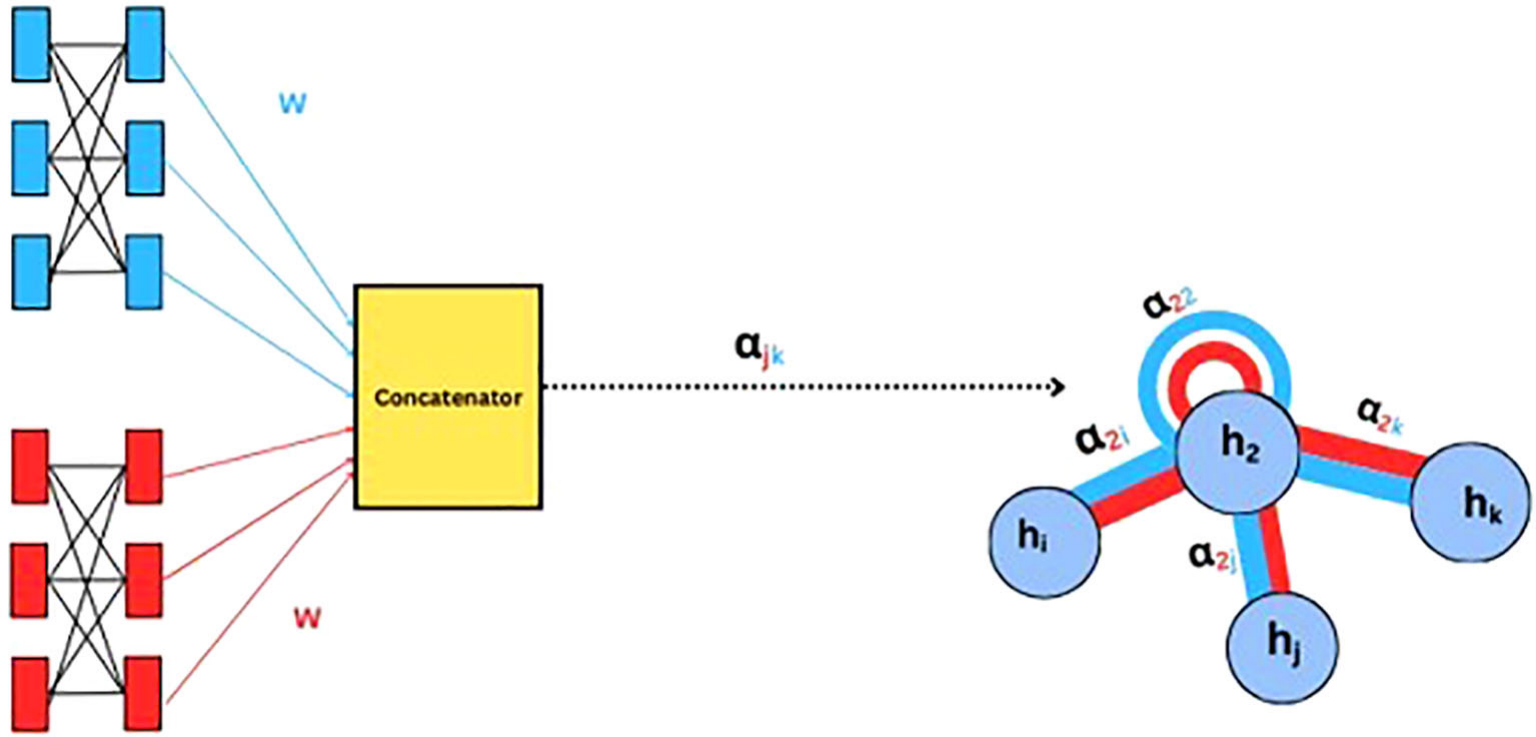
Schematic representation of the Graph Attention Network (GAT) architecture.

Graph Attention Networks (GAT) extend GCN by incorporating an attention mechanism (Velickovic et al., 2017). Instead of treating all neighbors equally, GATs compute attention scores between nodes by applying a shared linear transformation, followed by a dot product operation and a softmax normalization to determine the importance of each neighbor. The final node representation is obtained through a weighted sum of its neighbors’ features. This dynamic weighting enhances expressiveness, enabling GATs to handle varying neighborhood sizes and adapt to dynamic graph structures. Unlike traditional GCNs, which rely on predefined adjacency matrices, GATs can learn important relationships from data and hence improving the performance of node classification, graph classification, and link prediction. A sample illustration of how weight and attention are computed in GAT is shown in (**Figure 4**).

#### 3.3.5 GCN-GAT Hybrid architecture

The architecture combines Graph Convolution Networks (GCN) and Graph Attention Networks (GAT) to take advantage of the strengths of both models for processing graph-structured data. At the initial stage, the GCN layers capture local neighborhood information by aggregating and propagating node-level features through graph convolution, modeling spatial relationships within the graph. Thereafter, GAT layers use an attention mechanism to assign different weights to neighboring nodes, allowing the model to focus on the most relevant features, thus enhancing its feature representation.

**Figure 5:**
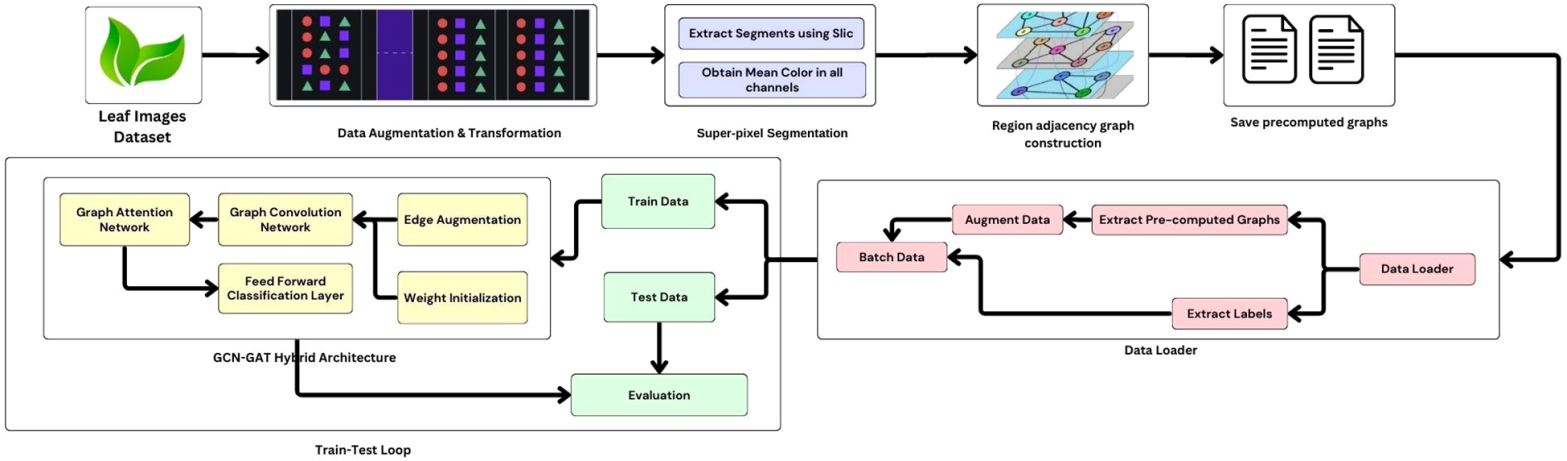
Proposed hybrid model: architecture and workflow.

In addition, a classifier layer with LeakyReLU activation is used to make the predictions of the leaf conditions based on the learned representations. **Figure 5** illustrates the workflow of the methodology. To increase the robustness of the model and to prevent overfitting, an augmentation strategy is used on the graph edges. This stochastic approach introduces randomness by randomly adding or removing edges in the graph during training, encouraging the model to generalize better and reducing its reliance on specific graph structures. The algorithm for edge augmentation is represented in **Algorithm 1**.

Algorithm 1 **Edge Augmentation.**

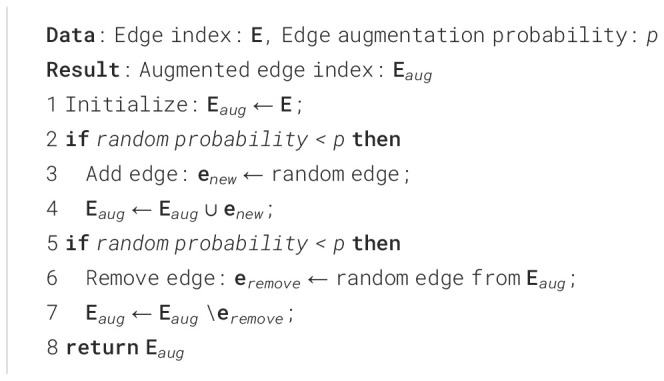

To address the challenges of vanishing gradients and promote more efficient training, the He initialization method has been adopted (He et al., 2015). This method helps maintain consistent variance in the activations of each layer, enabling faster convergence and improving gradient flow throughout the network. The He initialization can be mathematically expressed as shown in Equation 3.


(3) $$W∼U(-\sqrt{\frac{6}{n_{\text{in}}}},\sqrt{\frac{6}{n_{\text{in}}}})$$

Where,

- *W* represents the weight matrix.
- *n*_in_ is the number of input units (neurons) in the layer.
- The distribution is a uniform distribution, denoted by 𝒰, with bounds ± $\sqrt{\frac{6}{n_{\text{in}}}}$ .

### 3.4 Loss & evaluation metrics

Loss functions are mathematical functions used to measure how far predictions of the model deviate from the actual values. A higher loss indicates that the model is making more errors, while a lower loss suggests better predictive performance.

The architecture utilizes the cross-entropy loss function, which quantifies the difference between the predicted probability distribution and the true distribution.

#### 3.4.1 Cross-entropy loss

The cross-entropy loss function is widely used in classification problems to quantify the deviation between the predicted probability distribution and the actual distribution. It helps to optimize the model by penalizing incorrect predictions more heavily. The mathematical representation of the cross-entropy loss function is provided in Equation 4.


(4) $$\text{Cross–Entropy Loss}=-\sum_{i=1}^{N}\sum_{j=1}^{C}y_{ij}\log\;(p_{ij})$$

- y represents the true distribution of the labels.
-
$\hat{y}$ represents the predicted distribution of the labels.

Evaluation metrics are employed to assess the performance and quality of statistical or deep-learning models. To evaluate the hybrid model, the following metrics have been utilized:

#### 3.4.2 Accuracy

Accuracy represents the percentage of correctly classified leaf disease samples from the total number of leaf samples in the dataset. It measures the proportion of predictions that match the actual labels for each test image, reflecting the performance of the model in predicting the correct disease class label. Accuracy can be expressed as shown in Equation 5.


(5) $$\text{Accuracy}=\frac{Correct\;Predictions}{Total\;Predictions}\times 100$$

#### 3.4.3 Precision

Precision quantifies the reliability of the model when predicting a leaf as diseased. It specifically focuses on the cases where the model predicts the presence of a disease, evaluating how often these predictions are correct.

The formula for calculating precision is given in Equation 6.


(6) $$\text{Precision}=\frac{True\;Positives}{True\;Positives+False\;Positives}$$

#### 3.4.4 Recall

Recall measures the ability of the model to correctly identify all diseased leaves from the total number of leaves that actually have the disease. It highlights the capability of the model to capture as many true positives as possible. The formula for calculating the recall is presented in Equation 7.


(7) $$\text{Recall}=\frac{True\;Positives}{True\;Positives+False\;Negatives}$$

#### 3.4.5 F1-score

The F1-score combines precision and recall into a single metric, providing a balanced evaluation of the performance of the model for each disease class. This accounts for both the ability of the model to detect diseased leaves and its accuracy in making predictions.

The formula for calculating the F1-score is given in Equation 8.


(8) $$F1-score=\frac{2\times Precision\times Recall}{Precision+Recall}$$

#### 3.4.6 Confusion matrix

The confusion matrix provides a detailed breakdown of the predictions by the model, categorizing them into various outcomes. For each class, it shows the number of samples that are correctly or incorrectly classified. This provides a comprehensive view of the performance of the model, highlighting both its accurate predictions and the areas where misclassifications occur.


$$C=[\begin{array}{cc} TN & FP\\ FN & TP \end{array}]$$

Where, for each leaf disease class *i*, the confusion matrix terms are defined as follows in the multi-class classification context:

- *TP*: The number of leaf images correctly predicted as a member of class *i*.
- *FP*: The number of leaf images from other classes incorrectly predicted as class *i*.
- *FN*: The number of leaf images belonging to class *i* incorrectly predicted as other classes.
- *TN*: The number of leaf images correctly predicted as *not* belonging to class *i* (i.e., all other classes correctly identified as not *i*).

In multiclass classification, this matrix is extended such that each row and column corresponds to a class, providing a more granular evaluation of the performance of the model across all classes.

## 4 Results and discussions

The datasets were evaluated using three distinct model architectures: Graph Convolution Networks (GCN), Graph Attention Networks (GAT), and the hybrid architecture, GCN-GAT. The performance of these models was analyzed to understand their effectiveness in the detection of leaf disease across the different dataset. All models were trained for 100 epochs, and their performance was evaluated using standard evaluation metrics, namely Precision, Recall, F1-Score, and Accuracy, including the analysis of loss and accuracy curves. The key training parameters for the experiments (determined through hyperparameter tuning) are summarized in **Table 3**.

**Table 3:** Details of experimental setup and parameters.

Parameter	Settings
Image Size	(128, 128, 3)
Batch Size	32
Learning Rate	0.001
Optimizer	Adam
Attention Heads	2
Hidden Layers	512
GCN Layers	2
GAT Layers	2
Platform	Google Colab
GPU	NVIDIA A100

For the Apple Leaf dataset, the GCN model achieved an accuracy of 92.71%, with a precision of 0.9265, recall of 0.9271, and an F1-Score of 0.9267. In comparison, the GAT model attained an accuracy of 82.03%, with precision, recall, and F1-Score values of 0.8558, 0.8203, and 0.8202, respectively. The hybrid architecture (GCN-GAT) demonstrated exceptional performance, achieving an accuracy of 99.73%, along with a precision of 0.9974, recall of 0.9974, and an F1-Score of 0.9974. The performance results of the different models on the Apple Leaf dataset are summarized in **Table 4**.

**Table 4:** Comparative analysis of GCN, GAT, and hybrid model performance on the apple leaf dataset.

Model	Accuracy	Precision	Recall	F1-score	Average loss
GCN	0.9271	0.9265	0.9271	0.9267	0.1987
GAT	0.8203	0.8558	0.8203	0.8202	0.8102
**GCN+GAT**	**0.9973**	**0.9974**	**0.9974**	**0.9974**	**0.0143**

The bold values indicate the comparatively best performance achieved by the proposed hybrid model.

Based on these results, the models were further evaluated using the Potato Leaf dataset to assess their effectiveness. The GCN model attained an accuracy of 94.77% on this model, with a precision of 0.9506, recall of 0.9478, and an F1-Score of 0.9475. Interestingly, the GAT model slightly underperformed compared to GCN, attaining an accuracy of 92.33%, with a precision of 0.9298, recall of 0.9233, and an F1-Score of 0.9243. However, the hybrid GCN-GAT model once again stood out, delivering the highest performance with an accuracy of 98.11%, a precision of 0.9811, recall of 0.9811, and an F1-Score of 0.9811. The performance metrics for the models on the Potato Leaf dataset are presented in **Table 5**, highlighting the comparison between GCN, GAT, and the hybrid GCN-GAT model.

**Table 5:** Comparative analysis of GCN, GAT, and hybrid model performance on the potato leaf dataset.

Model	Accuracy	Precision	Recall	F1-score	Average loss
GCN	0.9477	0.9506	0.9478	0.9475	0.1447
GAT	0.9233	0.9298	0.9233	0.9243	0.3902
**GCN+GAT**	**0.9811**	**0.9811**	**0.9811**	**0.9811**	**0.0548**

The bold values indicate the comparatively best performance achieved by the proposed hybrid model.

Finally, the models were tested on the Sugarcane Leaf dataset, which posed unique challenges due to complex patterns present in the images. On this dataset, the GCN model achieved an accuracy of 64.63%, with a precision of 0.6745, recall of 0.6464, and an F1-Score of 0.6399. The GAT model experienced a notable drop in performance, yielding an accuracy of 48.83%, with precision, recall, and F1-Score values of 0.5360, 0.4884, and 0.4703, respectively. In contrast, the GCN-GAT hybrid model demonstrated its robustness, achieving a significantly higher accuracy of 91.03%, with a precision of 0.9124, recall of 0.9104, and an F1-Score of 0.9101. These results are summarized in **Table 6**.

**Table 6:** Comparative analysis of GCN, GAT, and hybrid model performance on the sugarcane leaf dataset.

Model	Accuracy	Precision	Recall	F1-score	Average loss
GCN	0.6463	0.6745	0.6464	0.6399	0.9301
GAT	0.4883	0.5360	0.4884	0.4703	1.2542
**GCN+GAT**	**0.9103**	**0.9124**	**0.9104**	**0.9101**	**0.2651**

The bold values indicate the comparatively best performance achieved by the proposed hybrid model.

**Figure 6:**
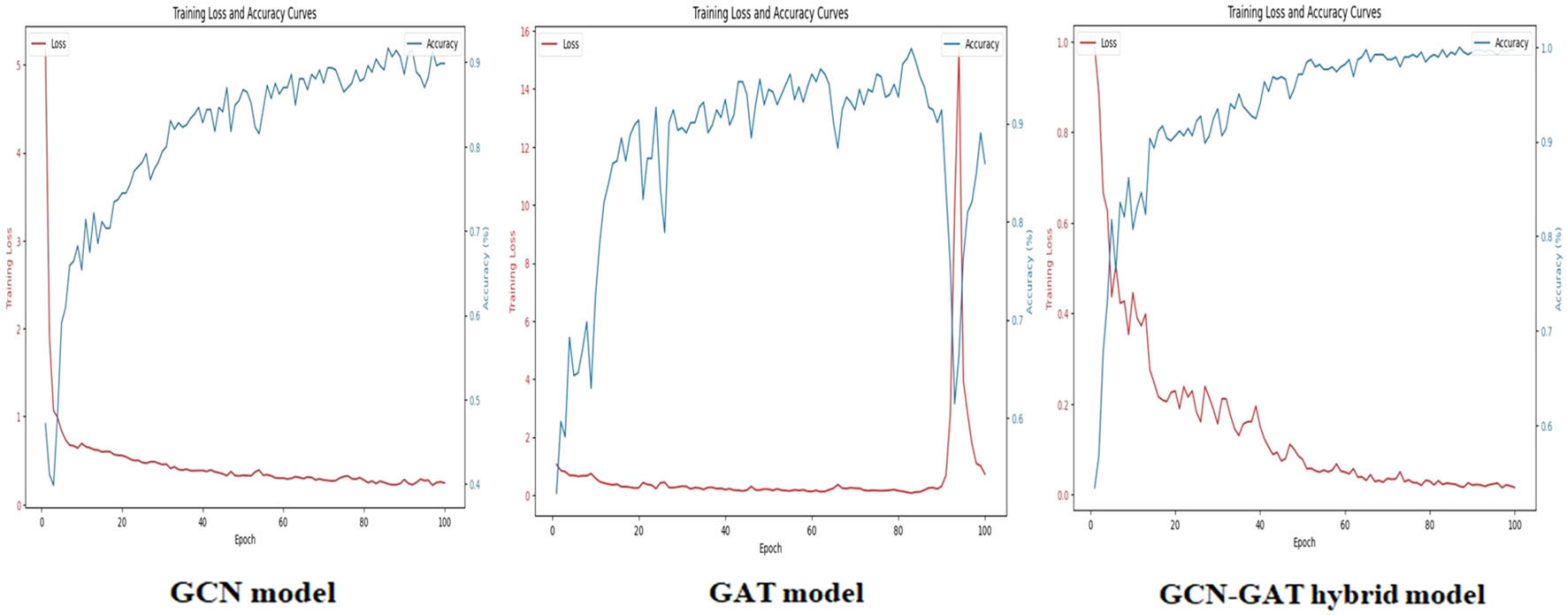
Training loss and accuracy curves for the GCN, GAT, and GCN-GAT hybrid models on the apple leaf disease dataset.

To evaluate training performance across different datasets, the loss and accuracy curves were examined. For the Apple Leaf Disease dataset, the GCN model recorded an average loss of 0.1987, whereas the GAT model showed a higher loss of 0.8102. The hybrid GCN-GAT architecture outperformed both, achieving a minimal loss of 0.0143, showcasing its superior learning capability. The corresponding loss and accuracy curves are depicted in **Figure 6**.

**Figure 7:**
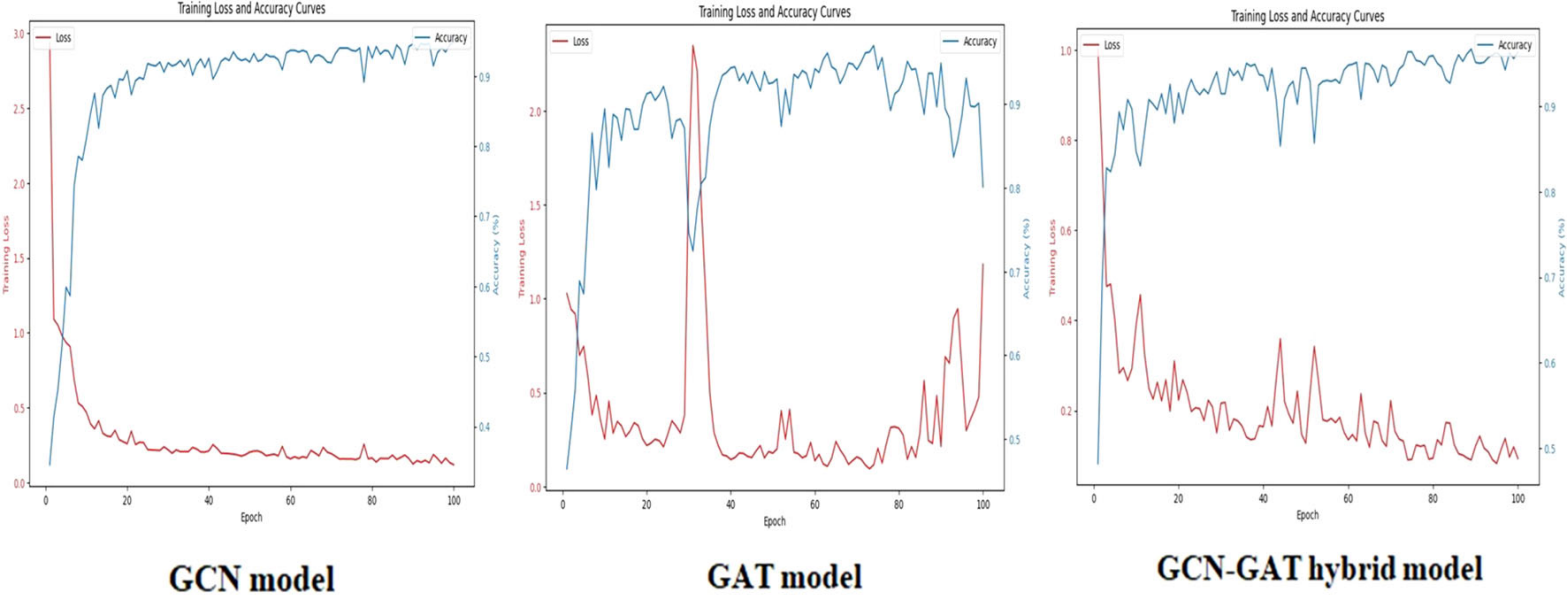
Training loss and accuracy curves for GCN, GAT, and GCN-GAT models on the potato leaf disease dataset.

On the Potato Leaf Disease dataset, the GCN model achieved a loss of 0.1447, while the GAT model demonstrated slightly higher efficiency with a loss of 0.3902. The hybrid architecture maintained its advantage, attaining the lowest loss of 0.0548. The respective training curves are illustrated in **Figure 7**.

**Figure 8:**
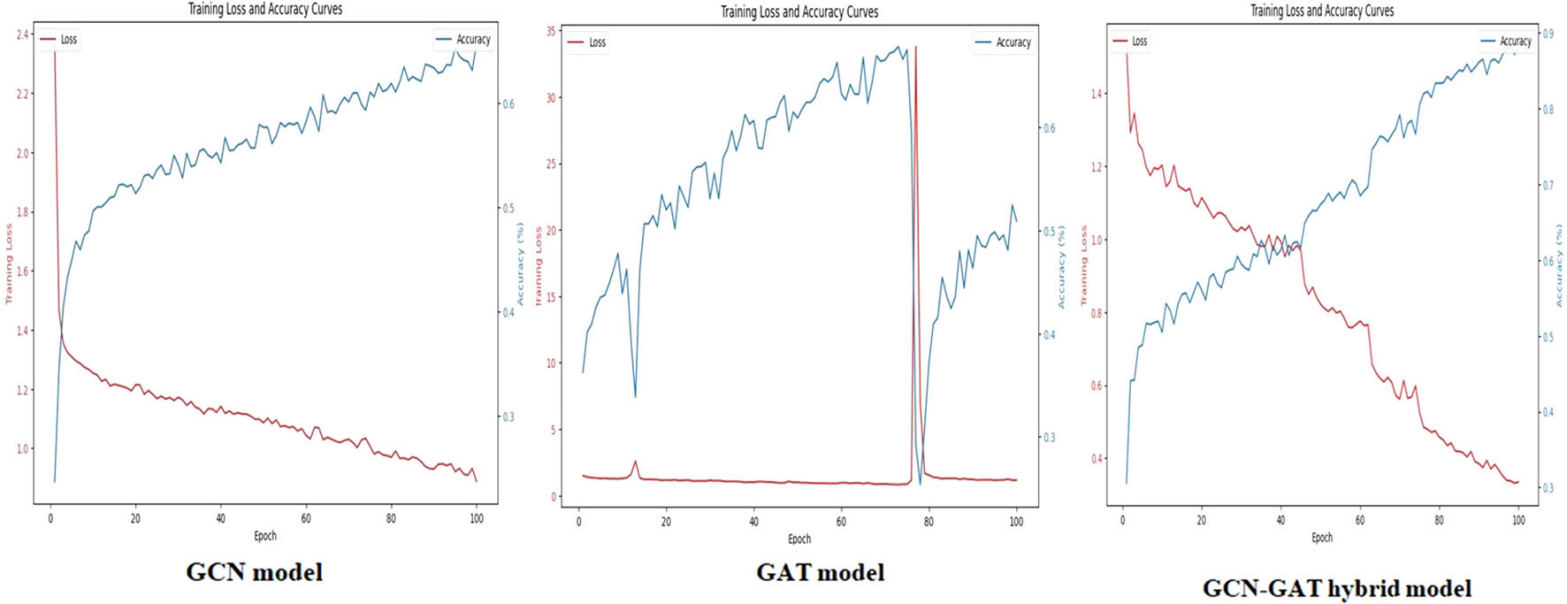
Training loss and accuracy curves for GCN, GAT, and GCN-GAT models on the sugarcane leaf disease dataset.

Similarly, for the Sugarcane Leaf Disease dataset, the GCN model experienced a loss of 0.9301, with the GAT model facing greater difficulty, recording a loss of 1.2542. The hybrid architecture once again proved to be the most effective, with a significantly reduced loss of 0.2651. The training performance curves for this dataset are presented in **Figure 8**.

To assess the classification performance per class in each dataset, the respective confusion matrices of individual architectures were inspected and analyzed.

**Figure 9:**
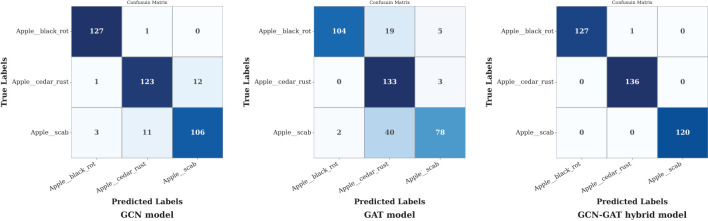
Confusion matrices of GCN, GAT, and hybrid GCN-GAT models for apple leaf disease classification.

In the apple leaf disease dataset, the GCN model effectively classified black rot apple leaves but misclassified 13 out of 136 samples in rust and 14 out of 120 in scab. The GAT model, however, underperformed across all classes, resulting in a higher frequency of misclassifications. In contrast, the GCN-GAT Hybrid model demonstrated superior performance, reducing misclassifications to 1 out of 128 in Black Rot leaf condition. The corresponding confusion matrices are shown in **Figure 9**.

**Figure 10:**
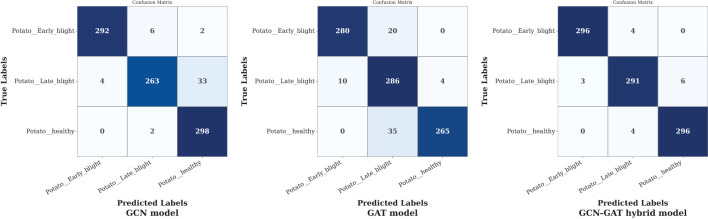
Confusion matrices of GCN, GAT, and hybrid GCN-GAT models for potato leaf disease classification.

On the potato leaf disease dataset, the GCN model misclassified 37 out of 300 samples in Late Blight conditions. Although the GAT model showed consistent performance across all classes, the GCN-GAT Hybrid model outperformed both, correctly classifying 883 out of 900 samples. The confusion matrices for each model are presented in **Figure 10**.

**Figure 11:**
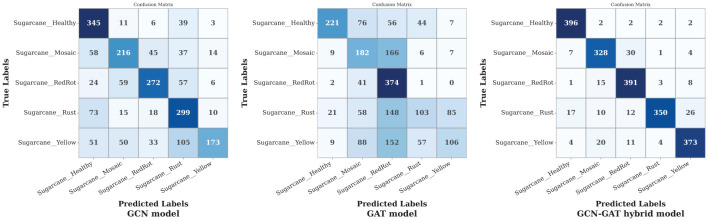
Confusion matrices of GCN, GAT, and hybrid GCN-GAT models for sugarcane leaf disease classification.

Finally, on the sugarcane leaf disease dataset, the GCN model exhibited varying performance across different classes, performing well in some but misclassifying more samples in others. The GAT model showed poor performance overall, making random predictions across classes. In contrast, the GCN-GAT Hybrid model, despite some misclassifications in Mosaic and Mosaic leaf conditions, outperformed both individual models. **Figure 11** presents the confusion matrix for the sugarcane dataset.

**Figure 12:**
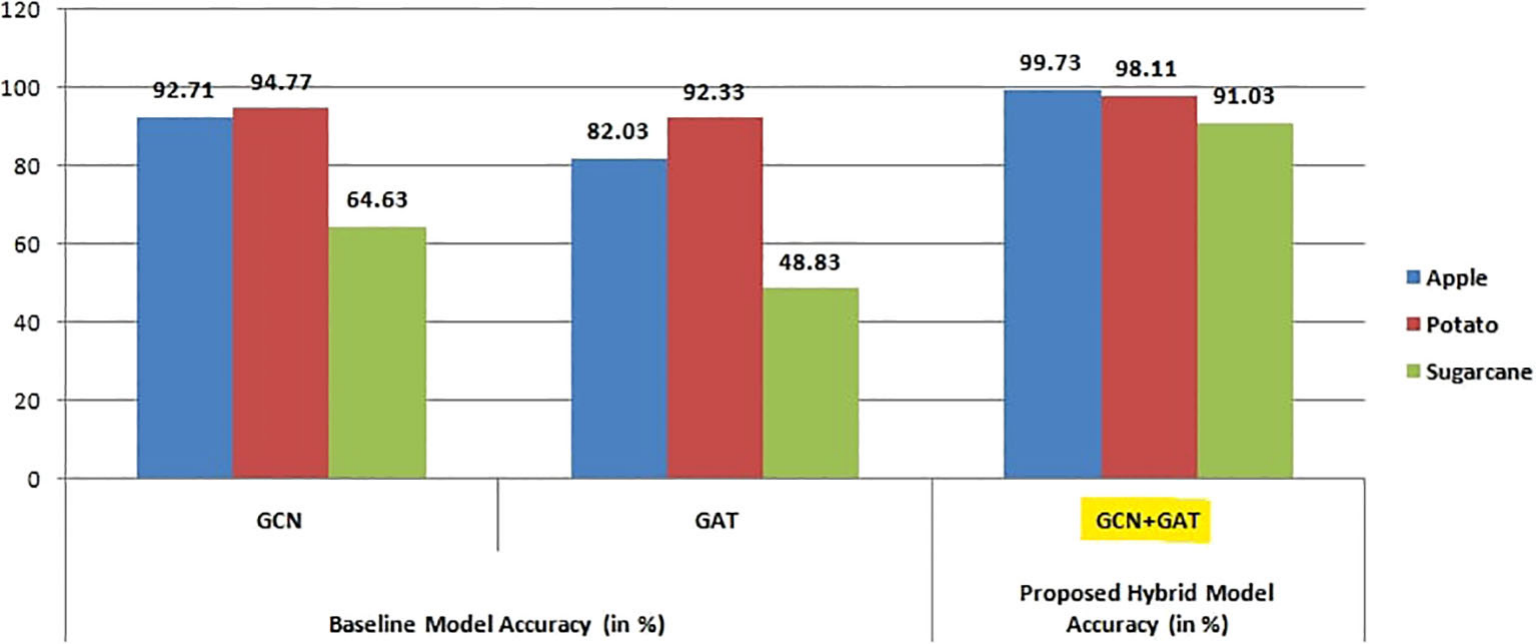
Visualization of accuracy comparison among GCN, GAT, and GCN+GAT models across apple, sugarcane, and potato leaf datasets.

**Figure 12** highlights the overall accuracy of all three architectures across multiple datasets, demonstrating the superior performance of the GCN-GAT hybrid model in consistently achieving higher classification accuracy compared to standalone GCN and GAT models.

To further evaluate the effectiveness of the proposed GCN-GAT hybrid architecture, we compare its performance with well-established state-of-the-art deep learning models commonly used for plant leaf disease classification. This comparison underscores the robustness and competitiveness of our approach across various datasets. The results are tabulated in **Table 7**.

**Table 7:** Performance comparison of state-of-the-art models for plant leaf disease classification.

Author(s) and year	Methodology	Dataset	Performance
(Zhong and Zhao, 2020)	DenseNet-121 based deep learning methods with regression, multi-label classification, and focal loss functions	Apple leaf dataset from AI-Challenger-Plant-DiseaseRecognition with 2,462 images across 6 disease classes	Achieved up to 93.71% accuracy on the test set
(Sholihati et al., 2020)	Deep learning with VGG16 and VGG19 architectures	Potato Leaf Disease Dataset consisting of 5 classes: Alternaria Solani, Healthy, Phytophthora Infestans, Virus, and Insect.	Achieved an average accuracy of 91.3%.
(Khalifa et al., 2021)	Deep CNN architecture with 14 layers	Potato Leaf Dataset with 3 classes (Healthy, Early Blight, Late Blight); augmented from 1,722 to 9,822 images	Achieved a mean testing accuracy of 98%
(Alsayed et al., 2021)	Transfer learning with pre-trained models (VGG16, ResNetV2, InceptionV3, MobileNetV2) for apple leaf disease classification	Apple leaf dataset including apple scab, cedar apple rust, and multiple disease classes	Achieved 94% accuracy with ResNetV2 and Adam optimizer
(Kumar and Patel, 2023)	The method detects multiple potato leaf diseases and alerts farmers via decision support system; pre-processing with median filtering and feature extraction using intuitionistic fuzzy local binary pattern	Potato leaf disease dataset with multiple disease classes including late blight, early blight, and more	Achieves approximately 96% accuracy based on reported graphs
(Daphal and Koli, 2024)	Attention-based Multilevel RCNN (AMRCNN)	The Sugarcane Leaf Disease Dataset contains 5 classes: Rust, Mosaic, Healthy, Red Rot, and Yellow Leaf	Achieved an accuracy of 86.53% on the dataset
(Wang et al., 2024)	Deep evidence fusion framework using multi-saliency maps and belief Cauchy–Schwarz divergence; EfficientNetV2-S backbone	Large combined apple leaf disease dataset from Northwest A&F University and AppleLeaf9 with multiple classes and augmented images	Achieved 98.1% accuracy with EfficientNetV2-S
-	Proposed GCN-GAT hybrid model combining spatial graph convolution and attention-based node representation	Apple leaf Potato leaf Sugarcane leaf	99.73% accuracy 98.11% accuracy 91.03% accuracy

## 5 Conclusion

An architecture using the GCN-GAT hybrid model was analyzed for leaf disease detection, leveraging the spatial feature-capturing capabilities of Graph Convolution Networks (GCN) and the feature prioritization strengths of Graph Attention Networks (GAT). The model was trained and evaluated on three diverse datasets: apple leaves (3 classes), potato leaves (3 classes), and sugarcane leaves (5 classes), representing varied leaf conditions. To highlight the superiority of the hybrid model, its performance was thoroughly analyzed and compared against the standalone GCN and GAT models.

The hybrid model consistently outperformed others across all datasets. On the apple dataset, it achieved a precision of 0.9974, recall of 0.9974, and an F1-score of 0.9974, with an average loss of 0.0143. For the potato dataset, the model achieved precision, recall, and F1-scores of 0.9811, 0.9811, and 0.9811, respectively, with an average loss of 0.0548. Despite the complexity of the sugarcane dataset, the model maintained robust results, achieving precision, recall, and F1-scores of 0.9124, 0.9104, 0.9101 each, with an average loss of 0.2651. These metrics underscore the adaptability and effectiveness of the hybrid model, with average accuracies of 99.74% for apple leaves, 98.11% for potato leaves, and 91.03% for sugarcane leaves.

The model when built in isolation using either GCN or GAT, a significant decrease in performance was observed across all the three datasets. On the apple dataset, the GCN model achieved a precision of 0.9443, recall of 0.9438, and an F1-score of 0.9436, with an average loss of 0.0345, while the GAT model achieved a precision of 0.9521, recall of 0.9519, and an F1-score of 0.9517, with an average loss of 0.0302. On the potato dataset, GCN recorded precision, recall, and F1-scores of 0.9134, 0.9129, and

0.9127, with an average loss of 0.0386, whereas GAT achieved precision, recall, and F1-scores of 0.9291, 0.9286, and 0.9284, with an average loss of 0.0331. For the sugarcane dataset, GCN and GAT showed relatively lower performance, with GCN achieving a precision of 0.8112, recall of 0.8108, and an F1-score of 0.8105 (average loss: 0.0564), and GAT achieving precision, recall, and F1-scores of 0.8293, 0.8290, and 0.8288 (average loss: 0.0517).

In summary, the GCN-GAT hybrid model significantly outperformed the standalone GCN and GAT models across all datasets, demonstrating its ability to effectively balance spatial feature extraction and feature prioritization. The inclusion of the idea of spatial information along with the prioritization of features enabled the hybrid architecture to achieve higher accuracy and lower loss, making it a robust solution for leaf disease classification.

Beyond current implementations, future work could focus on optimizing the computational efficiency of the hybrid model to facilitate its deployment on low-power devices. The integration of the proposed model into a real-time agricultural framework has the potential to revolutionize disease detection systems, ensuring healthier crops, reducing losses, and contributing to crop security.

## Data Availability

The datasets analyzed for this study can be found here: Sugarcane leaf disease dataset (Sankalana, 2022), potato leaf disease data set (Putra, 2020), and apple leaf disease dataset (Antor, 2020). The preprocessed and precomputed datasets can be obtained upon request from the corresponding author. Further inquiries can be directed to the corresponding author.
